# Insecticide Resistance in Fleas

**DOI:** 10.3390/insects7010010

**Published:** 2016-03-17

**Authors:** Michael K. Rust

**Affiliations:** Department of Entomology, University of California Riverside, Riverside, CA 92521, USA; michael.rust@ucr.edu; Tel.: +1-951-827-5327

**Keywords:** *Ctenocephalides felis felis*, *Ctenocephalides canis*, *Pulex irritans*, *Xenospylla cheopis*

## Abstract

Fleas are the major ectoparasite of cats, dogs, and rodents worldwide and potential vectors of animal diseases. In the past two decades the majority of new control treatments have been either topically applied or orally administered to the host. Most reports concerning the development of insecticide resistance deal with the cat flea, *Ctenocephalides felis felis*. Historically, insecticide resistance has developed to many of the insecticides used to control fleas in the environment including carbamates, organophosphates, and pyrethroids. Product failures have been reported with some of the new topical treatments, but actual resistance has not yet been demonstrated. Failures have often been attributed to operational factors such as failure to adequately treat the pet and follow label directions. With the addition of so many new chemistries additional monitoring of flea populations is needed.

## 1. Introduction

Adult fleas are ectoparasites of mammals and birds worldwide. Approximately, 2525 species have been identified, 96% infesting mammals and 4% infesting birds [1]. Several species are important because of the diseases that they transmit, especially some belonging to the genera *Ctenocephalides,* *Pulex*, and *Xenopyslla* [1,2,3,4]. Most of the control efforts and the use of insecticides have been directed at these species and this review will focus on them.

Fleas are the major insect ectoparasites of domesticated cats and dogs worldwide, several species being regularly collected [2]. The cat flea, *Ctenocephalides felis felis* (Bouché), is the most common and important pest species, but another subspecies exist. The subspecies *C. felis strongylus* (Jordan) is restricted to Saharan Africa and adjacent islands, but has been accidentally spread to wild animals in South Africa [5]. Two former subspecies of cat flea, *C. orientis* (Jordan) and *C. damarensis* Jordan have been elevated to full species status [6]. Molecular studies support the elevation of *C. orientis* to species status [7]. For brevity *C. felis felis* will be referred to as *C. felis*. The other commonly reported species are the dog flea, *Ctenocephalides canis* (Curtis); the sticktight flea, *Echidnophaga gallinacea* (Westwood); and the human flea, *Pulex irritans* L. The existence of morphological variations of characters used to differentiate *C. felis* and *C. canis* require that host data, geographical distribution, and the prevalence of infestations also be used in their determination [8]. *Pulex simulans* Baker is often mistaken for *P. irritans* and some records especially in the US may reflect this misidentification [1,9]. In regards to the potential development of insecticide resistance, the other species of flea that is important is the oriental rat flea, *Xenopsylla cheopis* (Rothschild).

In recent years a number of surveys of cats and dogs have been conducted worldwide. In Columbia, 94.2% of all fleas collected from cats and dogs were *C. felis*, and 5.8% were *P. irritans*, which were limited primarily to dogs [10]. Similarly, in western Hungary, 63% of the cats inspected had *C. felis*, and only 3.2% and 2.2% had *C. canis* and *P. irritans*, respectively [11]. Another survey in Hungary found fleas more prevalent on pets from agricultural areas (20.2%) than on pets in urban settings (12.0%). Dogs were infested with *C. felis*, *C. canis*, and *P. irritans* [12]. Of the 1938 fleas collected from 217 cats in Spain, 98.4% were *C. felis* and only 1.1% and 0.5% were *C. canis* and *P. irritans*, respectively [13]*.* In Albania, 75.7% of dogs were infested with *C. canis,* whereas 8.3% and 5.0% were infested with *C. felis* and *P. irritans*, respectively [14]. Cats were only infested with *C. felis*. In Thailand, 86.2% of dogs were infested with *C. orientis* and 1.1% were infested with *E. gallinacea* [15]. In a survey of rural and urban cats in Mexico, 53% were infested with at least one flea species and of the fleas recovered, 89% were *C. felis* [16]. About 34% of cats were infested with *C. canis*, but this is somewhat atypical because *C. canis* is rarely found on cats. *E. gallinacea* was reported on 14% of the cats. In Rio de Janeiro, 60% of the cats inspected had *C. felis* [17]. *C. felis* had more different hosts, a wider geographical range and more importance as a disease vector than *C. canis* in Brazil [8]. Dogs were surveyed on Robinson Crusoe Island, Chile, with *C. canis*, *C. felis* and *P. irritans* making up 45.6, 19.3 and 39.5% of the fleas collected, respectively [18]. Similarly, *C. canis*, *C. felis* and *P. irritans* were found on cats and dogs in southern Ethiopia [19]. In southern Iran, 13.8% of dogs surveyed had only *C. canis* and 1.5% had only *P. irritans* [20]. In Australia, 2,530 fleas were collected from 291 cats and dogs. Of these 98.8% were *C. felis* and 2.2% were *E. gallinacea* [21].

In recent years there has been an increase in the number and different classes of insecticides used to control fleas [2,22,23] with three reviews [24,25,26]. Unlike many situations in agricultural pest management, this is not in response to a failure of new products to control fleas or the development of insecticide resistance. Rather, it is due to the lucrative nature of the flea control market and to the consumer’s desire for extra convenience. “Resistance may be defined as ‘a heritable change in the sensitivity of a pest population that is reflected in the repeated failure of a product to achieve the expected level of control when used according to the label recommendation for that pest species’. Cross-resistance occurs when resistance to one insecticide confers resistance to another insecticide, even where the insect has not been exposed to the latter product [27].” On the other hand, susceptibility is defined as “the inability to withstand a pesticide at normal use rate [28]”. These terms will be used throughout the review.

A historical account of insecticide resistance in fleas deals mostly with *C. felis* [29]. Resistance Ratios (RR_50_) were typically less than 20-fold, but *C. felis* clearly showed resistance to multiple insecticides and some cross-resistance between carbaryl and organophosphates [30]. Since this comprehensive review, many flea control products have incorporated new chemistries such as neonicotinoids, phenylpyrazoles, semicarbazones, spinosyns, and isoxazolines. A more recent discussion about resistance in fleas [30] updated the status of resistance as available at the Arthropod Pesticide Resistance Database [31]. To date there are reports of resistance to organochlorine, carbamates, organophosphates, pyrethroids, and pyrethrins for *C. felis*, *P. irritans* and *X. cheopis*. In Madagascar, 32 of 36 populations of *X. cheopis* were resistant to deltamethrin [32]. Susceptibility was correlated to latitude, but not to longitude, insecticide used, or the date of sampling. Most of the 12 field-collected isolates of *C. felis* were significantly less susceptible to deltamethrin and permethrin compared with a DPIL strain tested in 2001 [33].

An initiative to monitor susceptibility of field-collected isolates of *C. felis* worldwide was initiated by Bayer Animal Health in 2001 [34,35]. Between January 2002 and December 2012, 1516 isolates obtained from cats and 770 isolates from dogs were collected. Of these, larval bioassays were conducted on 1373 isolates for susceptibility to imidacloprid. Isolates were collected in Australia, France, Germany, the UK and the USA. No isolates had an observed slope, LC_50_ or LC_95_ significantly different from the susceptible laboratory strains. Baseline values against adult cat fleas for imidacloprid were consistent with previously published data [33].

One of the major goals of monitoring flea populations is to detect insecticide resistance early, allowing for alternative treatments. The long-term goal of resistance management is to extend the longevity of the current host-targeted therapies [33]. Understanding and preventing the development of insecticide resistance will ensure a broad selection of veterinary products.

The goals of this review are to update the existing literature on the susceptibility of fleas to insecticides, encourage the monitoring of field-collected isolates of fleas, and stimulate interest in the maintenance of susceptible flea strains.

## 2. Methodology

Little attention has been given to the best methods of collecting samples of fleas from hosts and then propagating them in the laboratory. Typically adult fleas have been collected for testing and rearing. When adult female cat fleas are removed from the host, the oocytes begin to undergo degenerative changes within 72 h [36]. When adult *C. felis* are dislodged or leave the host, all males were dead within 2 days and females were inactive by day 3 [37]. Thus, adult fleas need to be transferred to a new host or tested within several days. Collecting *C. felis* eggs from infested hosts allows the researcher about 16–18 days before new adult fleas emerge and new hosts are needed. Egg collections have been conducted in many veterinary clinics without too much difficulty [38,39]. In this manner, over 1600 field-collected isolates of *C. felis* have been obtained for testing [34,38].

Maintaining field-collected isolates of fleas in the laboratory presents a challenge. Typically cats have served as the primary host for laboratory reared colonies of *C. felis*. The cats must be isolated so that flea eggs of each isolate can be collected and reared requiring additional space and resources. Rearing systems with mice have been proposed and could allow for flea colony maintenance in more limited spaces [40]. Artificial membranes have been used to feed adult *C. felis* and determine the systemic activity of insecticides [41,42,43,44], and the effects of blood source on fecundity and reproduction [45,46]. A method involving sedated mice has also been attempted to determine the systemic activity of insecticides against adult fleas [47]. Feeding fleas through membrane systems may be an alternative solution. Improvements in the use of artificial membranes and blood sources have been reported and might lead to more effective rearing [45]. Over the years several different bioassays have been utilized to test for insecticide resistance in fleas. Most of the studies have involved briefly confining adult fleas to treated filter paper and counting the subsequent adult mortality following the recommended WHO method [29,48]. Abiotic factors such as solvents, formulations, substrates, relative humidity, and temperature can greatly affect confinement tests and present challenges to replicating or comparing data [29,49,50]. The time of day, illumination, and the density of fleas did not affect the toxicity of test compounds [49]. In WHO type filter paper tests the toxicity of chlorpyrifos and permethrin increased as the RH increased [50]. To more closely simulate the effects of insecticides against adult fleas on carpets exposure tests have been conducted on disks of nylon fabric [51]. The concentrations of five insecticides that produced 50% and 95% kill at 24 h were determined for 7 field-collected isolates and 4 laboratory strains. Two field-collected isolates were tolerant to several insecticides including carbaryl, malathion, permethrin, and synergized pyrethrin.

One of the advantages of conducting topical applications to adult fleas is that a precise dose can be applied to each adult. Some 18 different insecticides against the DPIL strain of *C. felis* [52,53]. The findings provided a historical reference point for later topical testing [33]. Additional studies with many of the newer active ingredients is needed.

Larval bioassays have been used to determine the toxicity of insecticides including IGRs [54,55]. A larval bioassay was developed to test the susceptibility of cat flea isolates to imidacloprid [56]. One of the advantages of testing the larvae is that flea eggs can be directly collected in the field and then tested in the lab, eliminating the need to rear or maintain flea isolates in the laboratory. Once diagnostic doses are determined for a particular insecticide, as few as 40 flea eggs are necessary to determine if the isolate is susceptible [38,39]. Then, only those isolates of interest are propagated.

Most of the on-animal treatments are designed to kill adult fleas with the exception of the Juvenile Hormone Analogs such as methoprene and pyriproxyfen or the Chitin Synthesis Inhibitors such as lufenuron. One of the concerns in testing larvae to determine resistance ratios is how the data might relate to adult toxicity. Fipronil and imidacloprid were tested against larvae and adults of three laboratory strains and eight field-collected isolates [57]. Insecticides were applied by topical applications to adults and to the larval rearing media for the larvae. The toxicity of fipronil and imidacloprid ranged from 0.11 to 0.40 ng/flea against adults, providing Resistance Ratios (RR_50_) ranging from 0.11 to 2.21. Fipronil and imidacloprid in larval bioassays provided LC_50_s that ranged from 0.07 to 0.16 ppm and 0.11 to 1.75 ppm, respectively, with RR_50_s ranging from 0.11 to 1.75. Both adult and larval bioassays indicated that there was no reduced susceptibility to fipronil or imidacloprid in laboratory strains or field collected isolates.

A rapid assay for detecting esterases in cat fleas has been developed, but has not used to any great extent [58]. The use of organophosphates and carbamates to control fleas has declined dramatically since the late 1990’s. Biochemical assays have given way to molecular diagnostic tools. Using a rapid PCR-based diagnostic assay, Bass *et al.* [59] showed that L1014F and T929V mutations associated with pyrethroid resistance were common in both UK and USA flea populations. Widespread presence of these mutations in laboratory strains of cat fleas suggest that they arose many years ago. Brunet *et al.* [60] reported that *Rdl* gene mutation (targeting resistance to cyclodiene insecticides) was present as homozygous alleles of 6 cat flea strains susceptible to topically applied fipronil on hosts. It was also reported in most of 12 field-collected and 6 laboratory strains and had no discernable effect on responses to fipronil [33]. Bass *et al.* [61] found 8 of 9 strains had the A302S mutation with the frequency varying among the 8 strains. Resistance to cyclodienes is caused by a single change in A302 in the GABA receptor unit termed *Rdl* (resistance to dieldrin). It is known to confer varying levels of cross resistance to fipronil. In a preliminary survey in the UK, 50% of the fleas were homozygous for the S302 allele.

## 3. Product Failures

The initial response to product failures by practitioners and clients is often to attribute them to insecticide resistance. However, in recent years many of these failures have been ascribed to operational factors [62,63,64]. These include the failure to properly treat all pets in a household, to follow label instructions, to continue treatments in winter months, and to properly apply the product to the animal [63,64]. Many pets are left untreated. In Hungary, 51.4% of the pet owners with infested pets had not applied a treatment in the past year or more [12]. Some topically applied products are adversely affected by excessive bathing or shampoos [63].

A failure to properly treat pets may increase the likelihood of the pet becoming re-infested. Feral animals such as opossums and raccoons may serve as an outdoor reservoir and re-infestation of pets [63,65].

## 4. Resistance to New Oral and Topical Therapies

Since the introduction of the IGR lufenuron, fipronil and imidacloprid in the mid-1990s there has been little direct evidence of resistance developing to them. One isolate was reported to have low sensitivity to imidacloprid, but sensitive to fipronil [66]. Most of the focus has been on the susceptibility of cat fleas to fipronil and imidacloprid and the other new chemistries have not been tested. Extensive monitoring of nearly 1600 field-collected isolates have failed to show any decrease in susceptibility to imidacloprid [34]. Larval and adult bioassays of laboratory and field-collected isolates have shown some variability in response, but no significant reduction in their susceptibility to fipronil or imidacloprid [57].

Laboratory studies with certain field-collected isolates of *C. felis* have shown a variability in response to insecticides and reduced efficacy when treated dogs were challenged 28 days after being treated [30]. The KS1 strain has been frequently used in the evaluations of new therapies. It is an interesting strain of *C. felis* that was collected from a Kansas shelter in 1990 [30]. This strain likely has developed resistance to pyrethroids and possibly organophosphates as supported by molecular evidence and actual bioassays [30,33,59]. There is reduced susceptibility to fipronil, imidacloprid and spinosad compared with some strains which was most apparent when treated animals were challenged 28–30 days after the initial treatment [30]. Of three strains tested in a filter paper bioassay, KS1 was the most susceptible strain to fipronil and a strain from Lake Mary, FL, was most tolerant, the RR_50_ being 1.99 [67]. Topical applications of fipronil and imidacloprid to adult KS1 fleas were similar to other laboratory strains and field-collected isolates [33] indicating a broad range of susceptibility, but no resistance to either insecticide.

The laboratory and on-animal studies conducted with different strains of cat fleas indicate that there is variation in responses to insecticides. The LC_50_’s of 18 field-collected isolates of *C. felis* larvae had a range of 0.14 to 1.52 ppm of imidacloprid with many of the probit lines overlapping [38]. The range of responses is typically not reported because most laboratory and on-animal studies are usually conducted with only one *C. felis* strain.

The use of organophosphates, carbamates and pyrethroids to control cat fleas generated resistance within a short period of time. There are genetic, biological and operational factors that affect the development of resistance in insects [30,62]. Some of the biological factors that may be important in contributing to resistance in fleas are the rapid generation turn-over and the number of offspring per generation. Operational factors that might contribute to the development of resistance might include the chemical nature of the insecticide, persistence of residues, and the number of applications. However, the neonicotinoids and phenylpyrazoles have now been widely used for nearly 2 decades without a significant decrease in the susceptibility of *C. felis* to them. Are there any abiotic or biotic factors that might explain the lack of insecticide resistance developing to these new chemistries?

Factors that might contribute to the lack of resistance to the new chemistries are their mode of application and their activity against multiple life stages. First, topical applications to the surface of the pet or oral ingestion of drugs are designed to rapidly kill adult fleas and prevent them from feeding. Consequently, flea egg production and the adult fecal blood droplets necessary for larval development are abruptly disrupted. Secondly, on-animal treatments interrupt continuous flea development in the environment as demonstrated in many simulated home environment studies [68,69,70,71,72,73,74,75,76]. Insecticides capable of disrupting the life cycle in these studies include fipronil, fluralaner, imidacloprid, lufenuron, pyriproxyfen, and selamectin. Thus, one of the major sources of adult fleas for the pet is eliminated by preventing indoor breeding. Last, some of the treatments include insecticides with multiple modes of action. One study suggests that methoprene may synergize the activity of fipronil [77]. Another study shows that the IGR pyriproxyfen synergizes methoprene [78]. Interactions of insecticides with different modes of action against different life stages may delay the development of resistance.

## 5. Conclusions and Future Directions

One problem that has arisen in testing insecticide resistance in cat flea populations is the availability of a standard susceptible strain. Molecular studies [59,60,61] have shown that most laboratory “susceptible” strains do have the presence of resistant alleles for *Rdl* and the knockdown alleles. A collective effort to find populations of *C. felis* that are homozygous susceptible to all of the known suspected resistant alleles is needed. The strain needs to be maintained and made available to researchers and industry. For example, the Orlando normal strain of German cockroach, *Blattella germanica* (L), has served as an industry wide susceptible strain for decades.

New chemistries such as neonicotinoids, phenylpyrazoles, semicarbazones, spinosyns, and isoxazolines have been incorporated into oral and spot-on applications to control cat fleas. While very effective in controlling fleas on the host, very little is known concerning their toxicity to adult and larval fleas. Baseline data are needed to compare with field-collected populations.

Monitoring and surveillance of field-populations of fleas is essential if we are to be proactive in preventing insecticide resistance from developing. Vigilance is necessary if we are to protect and preserve our active chemistries for flea control.
